# The dual roles of exosomes in prostate cancer: mechanisms in tumorigenesis and avenues for clinical translation

**DOI:** 10.3389/fimmu.2026.1748272

**Published:** 2026-02-11

**Authors:** Mingyun Yu, Dan Zhou, Huijie Wei, Tong Wu, Jiahong Fan, Guanghe Ran, Chong Zhang

**Affiliations:** 1 Department of Pathology, Chongqing University Fuling Hospital, Chongqing, China; 2 Binhai New Area Hospital of Traditional Chinese Medicine (TCM), Fourth Teaching Hospital of Tianjin University of Traditional Chinese Medicine, Tianjin, China; 3 Chongqing Changshou Traditional Chinese Medicine Hospital, Chongqing, China

**Keywords:** cancer, clinical application, exosomes, mechanism, prostate cancer

## Abstract

Prostate cancer (PCa) management remains challenged by tumor heterogeneity, unpredictable progression, and limitations in early detection, driving demand for innovative biological insights. As pivotal mediators of intercellular communication, exosomes exhibit dualistic roles in PCa pathogenesis and therapy. While acting as ‘foes’ by facilitating epithelial-mesenchymal transition (EMT), angiogenesis, tumor microenvironment formation, metastasis, immune evasion, and therapy resistance, they concurrently serve as ‘friends’ through their diagnostic and therapeutic potential. Exosome-derived biomarkers enable non-invasive liquid biopsy for early diagnosis, risk stratification, and treatment monitoring. Moreover, engineered exosomes function as targeted drug carriers, delivering precision therapeutics to overcome treatment barriers. This review systematically examines exosomal biogenesis, isolation methodologies, and their bidirectional regulation in PCa progression, while exploring emerging diagnostic and therapeutic applications to advance exosome-mediated precision oncology.

## 1 Introduction

Prostate cancer (PCa) ranks as the second most prevalent malignancy and fifth leading cause of cancer mortality among males globally, posing a significant clinical challenge for prevention and treatment (1). Early-stage localized PCa can be effectively managed with surgery or radiation therapy, leading to favorable prognoses. However, once the disease progresses to advanced metastatic or castration-resistant stages (Castration-Resistant Prostate Cancer, CRPC), treatment options become significantly limited, and patient survival is severely compromised (2). Investigating the underlying mechanisms of PCa development, metastasis, and drug resistance is crucial for developing novel diagnostic and therapeutic strategies and improve the prognosis of patients (3). Accumulating evidence underscores exosomes—critical intercellular mediators—as pivotal yet complex regulators of prostate cancer progression, orchestrating tumor microenvironment (TME) dynamics while shaping clinical therapeutic strategies.

Exosomes are 30–150 nm nanovesicles released by cells upon multivesicular body (MVB) fusion with the plasma membrane into the extracellular space (4). They carry cell-specific “molecular cargo,” including proteins, lipids, DNA, mRNA, and non-coding RNAs with crucial regulatory functions, such as miRNA, lncRNA, and circRNA (5). These bioactive molecules internalize into recipient cells via endocytosis or membrane fusion, mediating intercellular communication and modulating both physiological and pathological processes (5). Refined exosome isolation and characterization methodologies—including differential ultracentrifugation, size exclusion chromatography, and immunoaffinity purification—enable rigorous investigation of exosomal functions across diverse pathologies, particularly cancer (6).

Exosomes exhibit a notable “double-edged sword” characteristic in the complex pathogenesis of prostate cancer. On one hand, as accomplices of the tumor, exosomes secreted by prostate cancer cells serve as key drivers of malignant progression. They transmit signals that induce epithelial-mesenchymal transition (EMT), enhancing the invasion and migration capabilities of tumor cells; promote angiogenesis, providing nutritional support for tumor growth and dissemination; facilitate cancer cell metastasis; and contribute to the establishment of an immunosuppressive microenvironment, aiding tumor cells in evading immune clearance (7). Moreover, exosomes mediate drug resistance such as chemotherapy and targeted therapy has become an important source of clinical treatment failure. On the other hand, exosomes also demonstrate promising potential as “beacons of hope.” Ubiquitous in biofluids (e.g., blood, urine), exosomes encapsulate tumor-specific molecular signatures, thus establishing their utility as liquid biopsy targets. These vesicles serve as non-invasive tools for prostate cancer diagnosis, risk stratification, prognostic evaluation, and therapeutic response monitoring. Engineered exosomes exhibit innate biocompatibility, barrier-penetrating capability, and target specificity, positioning them as ideal nanocarriers. Their capacity to encapsulate chemotherapeutics, nucleic acid drugs (e.g., siRNA), and other nanotherapeutics enables development of precision oncology strategies with enhanced efficacy (8).

Exosomes play an extremely complex dual role in the field of prostate cancer: it is not only a key promoter of malignant evolution of tumors, but also a revolutionary diagnostic and therapeutic tool with great potential. A deeper understanding of the complex signaling networks mediated by exosomes and their bidirectional roles in prostate cancer is crucial, not only for a more comprehensive understanding of the disease’s nature and overcoming clinical challenges such as metastasis and drug resistance, but also for paving the way toward the development of exosome-based non-invasive diagnostics, precise prognostic assessments, and targeted therapies. This review will systematically discuss the fundamental biological characteristics of exosomes and their isolation and identification methods, with a particular focus on their dual roles in prostate cancer progression (mechanisms promoting tumor progression and their diagnostic and therapeutic applications). It will also explore the current challenges and future directions in exosome-based diagnostic and therapeutic strategies, aiming to provide new perspectives and theoretical foundations for precision medicine in prostate cancer.

## 2 Biological characteristics and isolation identification of exosomes

**Figure 1:**
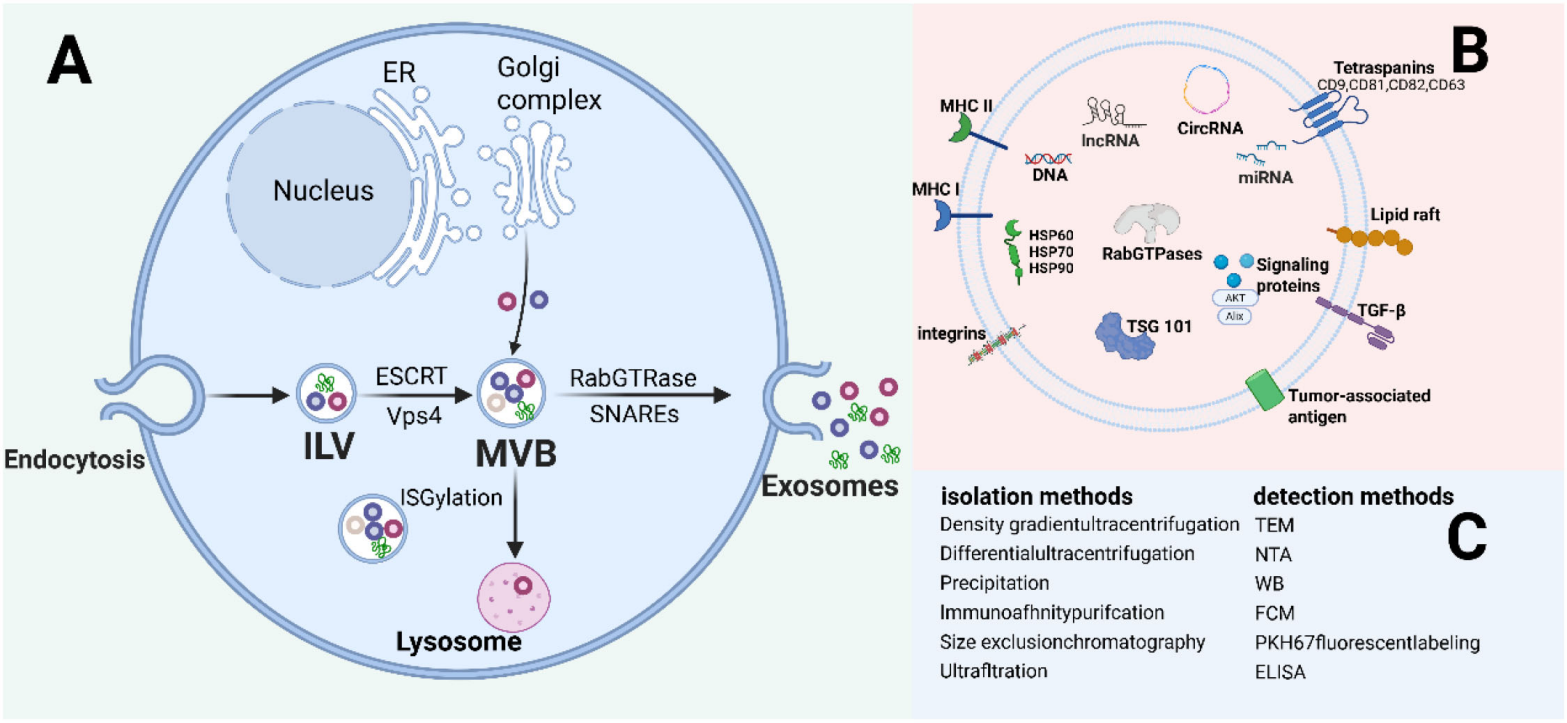
Exosome biogenesis, composition, and isolation/identification methods. **(A)** Exosomes originate through a conserved biogenic pathway initiated by donor cell plasma membrane internalization, forming early endosomes that progressively mature into late endosomes. During maturation, endosomal membranes undergo inward invagination to generate intraluminal vesicles (ILVs). These ILV-containing structures are designated multivesicular bodies (MVBs). Exosome release occurs upon MVB fusion with the plasma membrane, liberating ILVs into the extracellular space. **(B)** Common components of exosomes. DNA, RNA, four spanning proteins (CD9, CD63, CD81), PD-L1, integrin; Wnt proteins, ALIX, Syntenin, HSPs, GPC1, Rabs, Flotilin, etc. **(C)** Common isolation and identification methods of exosomes. Created with BioRender.com.

Exosomes are extracellular vesicles ranging from 30 to 150 nm in diameter. They are primarily composed of a lipid bilayer and contain a variety of biomolecules, including proteins, cellular metabolites, lipids, cytosolic components, ribonucleic acids, and nucleic acids (**Figure 1A**) (9). Exosomes can be secreted by nearly all cell types in the human body, such as stem cells, tumor cells, dendritic cells, macrophages, and other cell types (4). The specific origin of these exosomes can be determined by their surface ligands and receptors, playing key roles in intercellular communication and immune response modulation (10).

The body tightly regulates the biogenesis of exosomes. Exosomes are generated from the endosomal system (**Figure 1B**) (11). The formation of early endosomes (ILVs) is primarily due to the invagination of the cell membrane, leading to the formation of MVBs. During the transport of ILVs and the formation of MVBs, the endosomal sorting complex required for transport (ESCRT) plays a critical role (12). The ESCRT complex consists of ESCRT-0, ESCRT-I, ESCRT-II, ESCRT-III, and Vps4 (13, 14). ESCRT-0 is responsible for clustering ubiquitinated cargo proteins, while ESCRT-I/II assist in membrane curvature, ESCRT-III drives membrane scission, and Vps4 promotes the disassembly and recycling of the complex. Interestingly, several studies have shown that when ESCRT expression is inhibited, ILVs still form MVBs, suggesting that MVBs can also be generated through an ESCRT-independent pathway (15). Neutral sphingomyelinase 2 (nSMase2) generates ceramide, which promotes MVB invagination and ILV formation, and this pathway can be blocked by inhibitors like GW4869 to suppress exosome release (16). After MVB formation, they can either fuse with lysosomes or autophagosomes for degradation or merge with the plasma membrane via SNAREs and RabGTP proteins to release ILVs through exocytosis. Additionally, transmembrane proteins play a crucial role in this process. Numerous studies have indicated that transmembrane proteins such as CD9, CD69, CD81, CD82, CD61, heat shock proteins (HSP60, HSP70, and HSP90), tumor susceptibility gene 101 (TSG101), and integrins like ITGA3 and ITGB1 are involved in both ESCRT-dependent and -independent pathways of exosome biogenesis, thereby regulating cellular homeostasis (4, 17–20). However, the mechanisms and components of exosome biogenesis are modulated by different cell types (e.g., tumor cells, immune cells) and microenvironments (e.g., inflammation, hypoxia), displaying significant spatiotemporal and cell specificity, which warrants further investigation.

Exosomal cargo—encompassing proteins, lipids, and nucleic acids—mediates critical processes in cancer progression, immune regulation, and cardiovascular pathologies. These vesicles operate through two fundamental mechanisms: surface molecules directly activate intracellular signaling cascades upon binding target cell receptors, while membrane fusion enables cargo delivery to reprogram recipient cell function. Within tumor microenvironments, exosomes derived from tumor cells, immune cells (e.g., myeloid-derived suppressor cells [MDSCs]), and stromal cells critically influence disease dynamics by driving angiogenesis, enabling immune evasion, accelerating metastasis, and conferring drug resistance. Notably, such resistance manifests through chemoprotection or immunosuppression, thereby orchestrating tumor initiation, progression, and therapeutic responses (21). Crucially, beyond their pro-tumorigenic roles, exosomes also demonstrate significant potential as anti-cancer agents and therapeutic delivery platforms (22).

Exosomes are commonly found in various biological fluids, and their isolation and identification primarily rely on their physical properties—such as size and density—as well as specific immunological characteristics (**Figure 1C**) (23). Currently, widely used isolation techniques include differential ultracentrifugation, precipitation, immunoaffinity purification, size exclusion chromatography, and ultrafiltration (24). Differential ultracentrifugation is considered the “gold standard” and the most widely employed technique for exosome isolation, owing to its ability to process large sample volumes without the need for additional labeling, along with minimal contamination and relatively low operational cost. This method primarily includes density gradient centrifugation and differential centrifugation. However, it also has notable drawbacks, such as the requirement for expensive equipment, time-consuming procedures, and the potential to damage exosomal structure and integrity (25). Precipitation-based methods, in contrast, offer operational simplicity and high efficiency, and are suitable for a wide range of sample volumes, including both small and large-scale preparations. Despite these advantages, precipitation techniques suffer from significant limitations: they often co-precipitate non-specific contaminants, hinder accurate quantitative analysis of exosomal components, and are generally unsuitable for subsequent detailed characterization and functional studies of exosomes (26). Immunoaffinity purification-based purification leverages the specific binding between antibodies and antigens to isolate targeted exosome populations. While this approach allows for the selective capture of specific exosomes, it may also co-isolate non-specific vesicles and is generally unsuitable for large-scale sample processing (27). Size exclusion chromatography separates exosomes based on their hydrodynamic diameter, enabling high-purity isolation while preserving their structural integrity and biological activity. It is compatible with both small- and large-volume samples. However, the high cost of instrumentation and the inability to completely eliminate contaminants of similar size remain key limitations (28). Ultrafiltration utilizes membranes with defined pore sizes to concentrate and isolate exosomes, offering a cost-effective alternative to SEC. Nevertheless, membrane clogging can significantly reduce filter lifespan and affect reproducibility (29). Exosome identification typically relies on a combination of particle size, concentration, morphology, and surface markers (30). Commonly used characterization techniques include: Transmission electron microscopy (TEM) for assessing exosomal shape and size (31); Nanoparticle tracking analysis (NTA), which measures particle size and concentration based on light scattering and Brownian motion (32); Western blot, which quantifies exosomal protein markers (33); Flow cytometry, which enables quantitative analysis and surface marker-based sorting using fluorescent labeling (34); Additionally, PKH67 fluorescent labeling (35) and enzyme-linked immunosorbent assays (ELISA) (36) are also employed. Exosome isolation and identification strategies must align with experimental objectives and sample specifications. With ongoing technological advancements, exosome research continues to benefit from improvements in both isolation and identification methodologies, expanding their potential applications in biomedical science.

## 3 Exosomes in prostate cancer: promoting tumor progression

**Figure 2:**
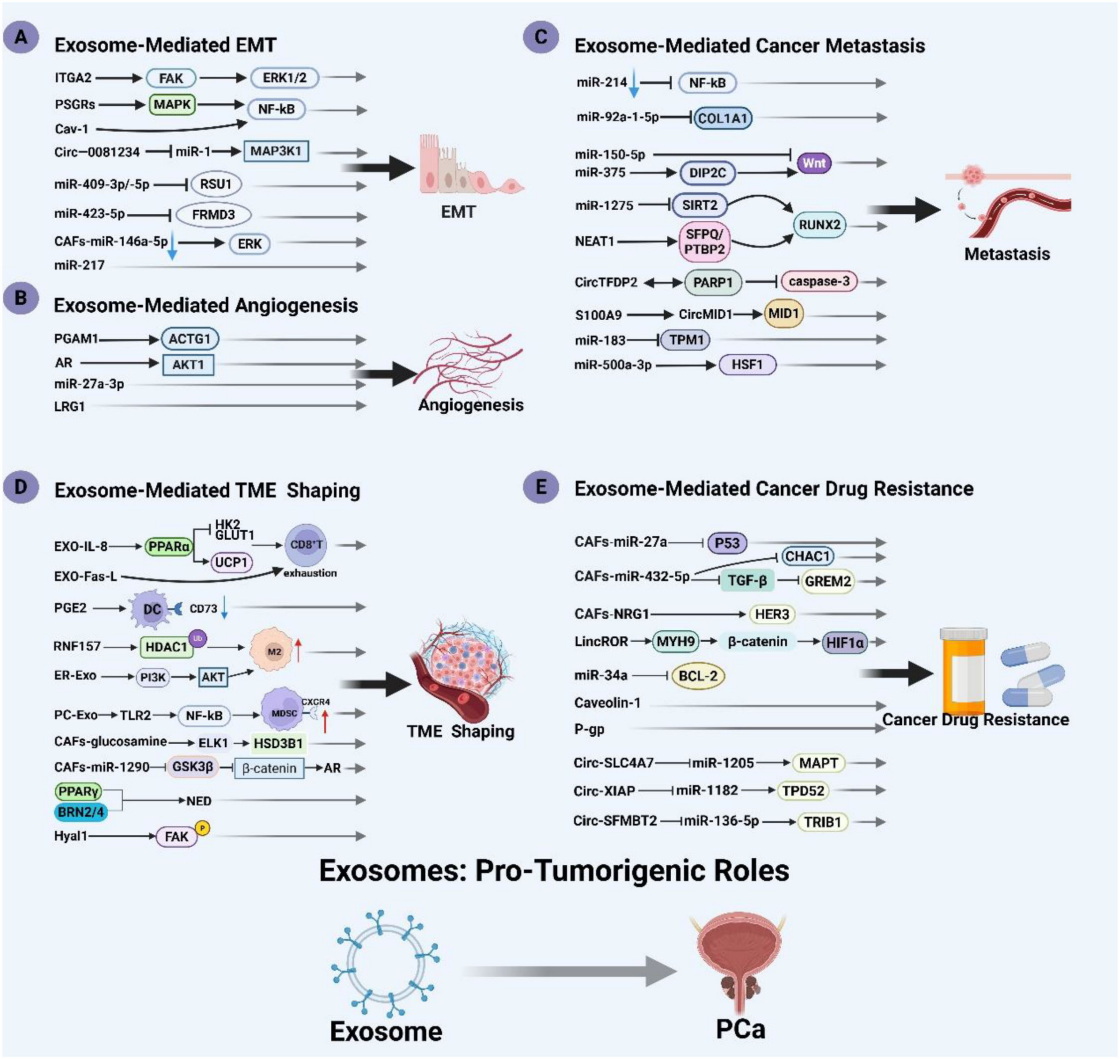
Exosomes in prostate cancer: promoting tumor progression. Exosomes promote prostate cancer by promoting EMT, angiogenesis, tumor microenvironment formation, immune escape, and antitumor drug resistance. The parts indicated in the diagram are: **(A)** [Exosome-Mediated EMT], **(B)** [Exosome-Mediated Angiogenesis], **(C)** [Exosome-Mediated Cancer Metastasis], **(D)** [Exosome-Mediated TME Shaping], **(E)** [Exosome-Mediated Cancer Drug Resistance]. Created with BioRender.com.

Acting as intercellular messengers, exosomes transfer biomaterials between cells. This process reprograms target cells, influencing their proliferation, survival, and immune surveillance. Released by virtually all cell types, including both benign and malignant prostate tissues, accumulating evidence indicates exosomes primarily exert detrimental effects in PCa (37). Here, we summarize the tumor-promoting effects of exosomes in PCa, including mechanisms such as EMT, angiogenesis, metastasis, tumor microenvironment formation, and the development of resistance to anticancer therapies (**Figure 2**).

### 3.1 Exosome-mediated epithelial–mesenchymal transition in prostate cancer

EMT is a process in which epithelial cells acquire mesenchymal characteristics during different cellular states, leading to reduced cell adhesion while gaining invasive and metastatic capabilities (38). Exosome-mediated EMT critically drives PCa progression.

Studies have found that the integrin α2 subunit (ITGA2) promotes cancer progression and metastasis. Exosomes derived from CRPC cells are enriched with ITGA2, which enhances focal adhesion kinase (FAK) and ERK1/2 activity in recipient cells, thereby promoting EMT. This process increases cell proliferation, migration, and invasion, ultimately driving the progression of PCa into a more aggressive form (39). Li et al. demonstrated that prostate-specific G protein-coupled receptor (PSGR)-bearing exosomes modulate bone metastasis-associated MAPK and NF-κB signaling through coordinated targeting of ICAM1, RELB, and IL1B, collectively driving EMT (40). Similarly, tumor-derived exosomes enriched with Cav-1 can induce neuroendocrine differentiation of PCa via the NF-κB signaling pathway, thereby promoting EMT (41). In addition, exosomes derived from PC3 cells carrying prostate-specific G protein-coupled receptors (PSGRs) promote EMT and stemness in low-invasive prostate cancer cells (LNCaP and RWPE-1), and also reshape the mRNA profile of LNCaP and RWPE-1 cells (42). Zhou et al. demonstrated *in vivo* and *in vitro* that serum exosomal miR-217 is significantly upregulated in PCa patients. This miRNA promotes tumor proliferation and invasion by modulating EMT markers—upregulating E-cadherin while downregulating vimentin (43). Josson et al. discovered that miR-409-3p/-5p in PCa exosomes can inhibit the expression of the tumor suppressor gene RAS suppressor 1 (RSU1), thereby promoting the EMT process (44). Exosome-derived circ-0081234 from cancer cells promotes EMT in PCa cells by inhibiting the expression of miR-1 and activating the MAP3K1 pathway (45). Wei et al. confirmed that exosomes carrying miR-423-5p in the bloodstream can target and inhibit FRMD3, thereby promoting EMT in PCa cells. This coordinated downregulation of E-cadherin with concomitant upregulation of N-cadherin and vimentin collectively promotes tumor proliferation, migration, and invasion *in vivo* (46). Under low androgen conditions, exosomes from cancer-associated fibroblasts (CAFs) isolated from PCa tissues exhibit a significant reduction in miR-146a-5p. This reduction further accelerates cancer cell metastasis by activating the EGFR/ERK axis through both *in vitro* and *in vivo* pathways, thereby enhancing EMT in PCa cells (47). These studies indicate that exosomes play a crucial role in mediating EMT and driving the progression of PCa.

### 3.2 Exosome-mediated angiogenesis

Angiogenesis generates new vascular networks to deliver oxygen and nutrients, enabling tumor growth and metastasis. Research shows that exosomes secreted by tumor cells constitute a primary angiogenesis-inducing mechanism.

In the context of various PCa hormone therapies, researchers have found complex interactions between androgen receptor (AR) signaling and exosome-mediated communication from PCa cells. These interactions enhance key factors in exosomes, such as AKT1, CALM1, PAK2, and CTNND1, which in turn stimulate tumor cell proliferation, migration, and angiogenesis (48). DeRita et al. demonstrated that PCa-derived exosomes carry elevated Src, insulin-like growth factor 1 receptor (IGF-IR), and FAK, driving angiogenesis (49). Under hypoxic conditions, prostate cancer-derived exosomes drive angiogenic processes by stimulating matrix metalloproteinase (MMP2/MMP9) activity and remodeling key extracellular matrix components (fibronectin, collagen), ultimately inducing vascular leakage that facilitates circulating tumor cell invasion (50). In addition, studies have shown that exosomal miR-27a-3p derived from PC-3 cells may participate in the angiogenesis process of CRPC (51). Liu et al. discovered that exosome-derived leucine-rich α2-glycoprotein 1 (LRG1) is involved in angiogenesis in PCa (52). Exosomal phosphoglycerate mutase 1 (PGAM1) promotes angiogenesis and invadopodia formation by interacting with ACTG1, thus initiating PCa cell metastasis and serving as a potential liquid biopsy marker for PCa metastasis (53).

Although angiogenesis plays a critical role in PCa, clinical studies of PCa have shown that anti-angiogenic therapies have failed to provide the expected clinical benefits and, instead, have increased toxicity, thereby promoting cancer progression (54). Current anti-angiogenic therapies for PCa demonstrate limited efficacy. The cited studies establish exosomes as pivotal mediators of PCa progression, particularly through neovascularization promotion, positioning them as promising therapeutic targets. Exosome-based strategies thus enable novel approaches for angiogenesis-targeted PCa treatment.

### 3.3 Exosome-mediated cancer metastasis

Approximately 90% of cancer-related deaths in humans are attributable to metastasis (55). Most malignant tumors are characterized by high invasiveness and a strong propensity for metastasis. The metastatic process involves a series of steps, including the invasion of primary tumor cells, survival within the circulatory system, and the subsequent adhesion to and colonization of distant organs (56, 57). Enhanced migratory capacity and immune evasion of cancer cells are key determinants driving these processes. Metastatic PCa commonly spreads to various organs, including bone, liver, lungs, and lymph nodes. Exosomes, as pivotal mediators of intercellular communication, play critical roles throughout multiple stages of tumor metastasis.

The skeleton is a common target organ for PCa metastasis. Tumor growth in bone arises from tumor-bone crosstalk that dysregulates physiological bone homeostasis. This balance, governed by osteoblast-mediated formation and osteoclast-driven resorption, establishes a pre-metastatic niche. Accumulating evidence confirms exosomal mediation of communication between PCa cells and the bone metastatic microenvironment. Yu et al. demonstrated that exosomal miRNA-92a-1-5p derived from PCa cells disrupts the balance between osteoblasts and osteoclasts by directly targeting and suppressing COL1A1, thereby promoting osteoclast differentiation while inhibiting osteoblastogenesis. This imbalance accelerates degradation and remodeling of the bone extracellular matrix (ECM) and facilitates the formation of a pre-metastatic niche, creating favorable conditions for PCa bone metastasis (58). Yang et al. further demonstrated that PC-3 cell-derived exosomes suppress osteoclast differentiation via miR-214 downregulation and NF-κB pathway blockade, enhancing PCa infiltration at bone metastatic sites (59). Concurrently, PCa exosomal miR-375 activates Wnt signaling by targeting DIP2C, driving osteoblastic metastasis (60). Additionally, exosomal miR-1275 upregulates RUNX2 (a key osteoblast regulator) through SIRT2 inhibition, promoting osteoblast proliferation and function to accelerate PCa bone metastasis (61). Interestingly, NEAT1 critically regulates osteogenic differentiation in PCa-associated human bone marrow-derived mesenchymal stem cells(hBMSCs). PCa-derived exosomal NEAT1 transfers to hBMSCs, where it competitively sequesters miR-205-5p and modulates the SFPQ/PTBP2 axis. This cascade upregulates RUNX2, inducing osteogenic differentiation (62). Through osteogenic assays (alkaline phosphatase activity, matrix mineralization, and osteogenic markers), Li et al. confirmed that LNCaP-derived exosomal miR-375 potently promotes osteoblastic activity (63). Additionally, exosomal miR-150-5p may facilitate PCa bone metastasis, primarily through inhibition of the Wnt signaling pathway, as suggested by Gene Ontology (GO) and KEGG pathway analyses (64). Multiple studies have also reported that exosomal miR-409 and miR-141 derived from PCa cells can target the small heterodimer partner (SHP), contributing to bone metastasis (65, 66). Collectively, these findings indicate that exosomes can disrupt the homeostatic balance between osteoblasts and osteoclasts, thereby influencing the metastatic progression of PCa.

Moreover, additional studies have demonstrated that exosomes play a pivotal role in mediating PCa metastasis. Ding et al. demonstrated *in vivo* and *in vitro* that exosomal circTFDP2 binds PARP1 at its DNA-binding domain, inhibiting caspase-3-dependent PARP1 cleavage. This suppression reduces DNA damage in PCa cells, ultimately promoting tumor proliferation and metastasis (67). Gao et al. identified S100A9-enriched exosomes from MDSCs as key regulators that upregulate circMID1 in PC3 cells through miR-506-3p sponging, thereby promoting tumor cell proliferation, invasion, and migration (68). Han et al. further demonstrated that engineered exosomes targeting SIRT6 effectively inhibit the metastatic potential of PCa cell lines (69). Additionally, silencing of exosomal carbonic anhydrase I (CA1) in PC3 cells enhances tumor cell migration and invasion (70). Dai et al. found that exosomal miR-183 from PCa cells promotes cancer cell invasion and migration by downregulating TPM1 expression (71). Under hypoxic conditions, cancer-associated fibroblast (CAF)-derived exosomal miR-500a-3p, isolated from PCa tissues, promotes PCa metastasis by suppressing FBXW7 expression and upregulating HSF1, suggesting that miR-500a-3p may serve as a promising therapeutic target for metastatic PCa (72). Comparative RNA-seq of CAF- versus normal fibroblasts-derived exosomes from PCa/adjacent tissues identified differentially expressed miRNAs. Specifically, CAF-exosomal miR-1290 promotes PCa progression by suppressing GSK3β/β-catenin signaling, highlighting its therapeutic potential (73). Separately, Fabbri et al. showed that exosomal miR-21/29a activate TLR7/8 in immune cells, triggering pro-metastatic inflammation that facilitates tumor dissemination (74).

Collectively, accumulating evidence reveals exosomes as pivotal mediators of PCa metastasis through multifaceted molecular pathways. Vesicular cargo—particularly circRNAs, miRNAs, and proteins—orchestrate signaling cascades that drive malignant behaviors (proliferation, invasion, migration) while simultaneously remodeling tumor microenvironments and pre-metastatic niches. These insights fundamentally advance our comprehension of exosome-driven intercellular communication in PCa progression, extending beyond mechanistic understanding to reveal promising diagnostic biomarkers and therapeutic targets against metastatic disease.

### 3.4 Exosome-mediated tumor microenvironment remodeling and immune evasion in prostate cancer

The TME encompasses the dynamic cellular and molecular milieu surrounding neoplastic tissue. It is primarily composed of neighboring blood vessels, stromal cells (e.g., fibroblasts and neuroendocrine cells), immune cells (e.g., T lymphocytes, natural killer (NK) cells, dendritic cells (DCs), and macrophages), adipose-derived stem cells, various signaling molecules, and the extracellular matrix (ECM). It represents a complex and continuously evolving biological process. This evolving biological ecosystem facilitates complex intercellular communication in PCa through ECM remodeling, metabolic reprogramming, immune suppression, and angiogenesis. Based on the TME features that facilitate tumor initiation, survival, and metastasis, three major subtypes are recognized: hypoxia, inflammation, and immune suppression. Exosomes orchestrate these processes by establishing multifaceted communication networks. During TME reprogramming, exosomes serve as critical vectors for long-distance transport of bioactive cargo. In advanced malignancies, bidirectional crosstalk between the TME and tumor-derived exosomes drives proliferation, confers therapy resistance, and enables metastatic dissemination (75). As master regulators of intercellular signaling, exosomes play pivotal roles in PCa TME development.

Under hypoxic conditions, exosomes derived from LNCaP and PC3 prostate cancer cells modulate adherens junction protein expression. This remodeling enhances tumor cell aggressiveness and stemness while inducing microenvironmental alterations, collectively driving PCa progression (76). Abd Elmageed et al. demonstrated that the PCa microenvironment induces tumorigenic transformation of patient-derived adipose-derived stem cells (ADSCs). Mechanistically, this neoplastic reprogramming is mediated by PCa cell-secreted exosomes carrying specific microRNAs (miR-125b, miR-130b, and miR-155). These findings provide novel mechanistic insights into molecular drivers of PCa progression (77). Upregulated hyaluronidase 1 (Hyal1) in prostate tumor cells accelerates vesicular trafficking to potentiate stromal cell migration. Mechanistically, exosome-transferred Hyal1 executes its pro-tumorigenic functions by augmenting cellular adhesion to type IV collagen substrates, facilitating dynamic membrane clustering of β1 integrins, and propagating FAK phosphorylation. This concerted mechanism drives Hyal1-dependent PCa progression through extracellular matrix remodeling and motility activation (78).

Neuroendocrine cells drive disease progression through autocrine/paracrine secretion of peptide hormones and growth factors. Hormone-treated prostate cancers frequently develop neuroendocrine differentiation (NED), a phenotype associated with therapeutic resistance and poor survival. Exosomes derived from PC3 cells—with or without growth hormone-releasing hormone (GHRH) preconditioning—induce NED in LNCaP cells, evidenced by increased neurite-bearing cell populations, elevated neuron-specific enolase (NSE) expression, and enhanced proliferative/adhesive capacities. Notably, exosomes from GHRH-primed PC3 cells accelerate LNCaP proliferation more rapidly than those from untreated controls (79). Under IL-6 stimulation or androgen-deprived conditions, exosomal adipocyte differentiation-related protein (ADRP) from DU145 and LNCaP prostate cancer cells induces NED in CRPC through paracrine activation of peroxisome proliferator-activated receptor γ (PPARγ), a master adipogenic transcription factor (80). Notably, Bhagirath et al. demonstrated that exosomes from enzalutamide-resistant PCa models (LNCaP, Du145, PC3, C42B, NCI-H660) drive oncogenic lineage plasticity toward neuroendocrine states via release of neural transcription factors BRN2 and BRN4 (81).

The TME harbors abundant yet functionally impaired immune cells that facilitate tumor immune evasion. Mounting evidence indicates tumor-derived exosomes mediate immunosuppressive reprogramming of immune cells to establish a pro-tumorigenic niche in PCa. CD8^+^ T-cell depletion constitutes a major barrier to immunotherapy efficacy. Specifically, LNCaP-derived exosomes enriched with Fas ligand (FasL) induce apoptosis in CD8^+^ T lymphocytes (82). Xu et al. further revealed that PCa-derived exosomes shuttle IL-8 to suppress CD8^+^ T-cell function. Mechanistically, exosomal IL-8 hyperactivates PPARα in recipient cells, impairing glucose utilization through GLUT1/HK2 downregulation. This PPARα activation upregulates UCP1, redirecting fatty acid catabolism from ATP synthesis toward thermogenesis. This metabolic rewiring induces CD8^+^ T-cell bioenergetic crisis and functional exhaustion, facilitating immune escape (83). Parallel immune evasion occurs through NKG2D receptor modulation. As a critical cytotoxic receptor expressed on NK and CD8^+^ T cells, NKG2D plays pivotal roles in antitumor immunity. Mali et al. revealed that exosomal NKG2D ligands on human PCa cells selectively downregulate NKG2D expression in a dose-dependent manner. This ligand-mediated cis-regulation compromises cytolytic function of both NK and CD8^+^ T cells, promoting immunosuppression and tumor evasion (84). Salimu et al. further revealed that PCa -derived exosomes impair dendritic cell (DC) capacity for tumor antigen cross-presentation. Building on prior observations of these exosomes suppressing IL-2 secretion in CD4^+^ T cells and inducing ecto-5’-nucleotidase (CD73) surface expression on DCs, mechanistic studies identified exosome-borne prostaglandin E2 (PGE2) as the principal mediator of CD73 upregulation. This PGE2-CD73 axis critically disrupts DC functionality, providing novel insights into exosome-facilitated immune evasion through DC reprogramming (85). Cellular studies by Han et al. revealed that RNF157 mRNA within prostate cancer-derived exosomes is transported to macrophages, where its translation product binds histone deacetylase 1 (HDAC1). This interaction promotes HDAC1 ubiquitination, triggering ubiquitin-proteasomal degradation. The resultant HDAC1 depletion culminates in macrophage repolarization toward an M2-like phenotype. *In vivo* validation established that exosomal RNF157 accelerates prostate tumor growth through TAM-mediated M2 polarization in xenograft models (86). Under endoplasmic reticulum (ER) stress, prostate cancer cell-derived exosomes drive macrophage polarization toward an M2-like phenotype. This reprogramming involves exosomal transmission of stress signals that activate the PI3K/Akt pathway in macrophages. Akt-mediated signaling upregulates M2-associated genes (e.g., CD206, PD-L1) while suppressing CD16 (FcγRIII) expression, resulting in immunosuppressive functional conversion. These polarized tumor-associated macrophages (TAMs) secrete immunosuppressive factors that blunt antitumor immunity, facilitating tumor immune evasion, invasion and metastasis of tumor cells (87). Concurrently, PCa exosomes modulate MDSCs—key immunosuppressive populations that dampen immune effector responses. Li et al. demonstrated that tumor exosomes activate TLR2/NF-κB signaling in MDSCs, markedly upregulating surface CXCR4 expression. Enhanced CXCR4 levels potentiate MDSC chemotaxis toward CXCL12 gradients in the TME via CXCR4-CXCL12 axis-driven migration. Critically, pharmacological TLR2 inhibition using C29 antagonist significantly attenuated CXCR4 expression and impaired MDSC trafficking, confirming TLR2/NF-κB’s pivotal role in myeloid cell recruitment (88). Research indicates that CAFs, derived from fibroblasts and mesenchymal stem cells, promote tumor growth (89). Transforming growth factor-β (TGF-β) plays a critical role in the generation and maintenance of CAFs (90). For instance, exosomes derived from PCa cell lines (LNCaP, DU145, and PC3) exhibit surface enrichment of TGF-β. This TGF-β can activate SMAD3-associated signaling pathways, thereby inducing the acquisition of a CAF phenotype (91).

Furthermore, studies by Cui et al. demonstrate that exosomes originating from CAFs deliver glucosamine to PCa cells. Within the recipient cancer cells, this glucosamine elevates the levels of O-linked β-N-acetylglucosamine modification (O-GlcNAcylation) (92). O-GlcNAcylation, a crucial post-translational modification involving the attachment of β-N-acetylglucosamine to serine/threonine residues, modulates cellular nutrient sensing and stress responses. This modification subsequently enhances the transcriptional activity of the transcription factor ELK1. Activated ELK1 upregulates the expression of HSD3B1, the rate-limiting enzyme in steroid synthesis (93). The increased HSD3B1 expression stimulates *de novo* androgen synthesis within the tumor cells, activating the androgen receptor (AR) signaling pathway. Ultimately, this cascade drives the progression of CRPC (93). Wang et al. demonstrated a significant elevation in miR-1290 levels within exosomes derived from CAFs in PCa tissue. These CAF-secreted exosomes deliver miR-1290 to PCa cells, enhancing their migratory and invasive capacities and inducing EMT. Mechanistic investigations revealed that exosomal miR-1290 targets glycogen synthase kinase 3β (GSK3β), downregulating its expression and consequently impairing the GSK3β/β-catenin signaling axis. Specifically, the suppression of GSK3β expression diminishes β-catenin degradation, leading to its accumulation in the cytoplasm and nucleus. This accumulated β-catenin activates the transcription of downstream pro-metastatic genes, ultimately promoting an aggressive tumor phenotype (73).

In summary, exosomes critically orchestrate the PCa tumor microenvironment and enable immune evasion. Deciphering their molecular mechanisms in mediating tumor-immune cell crosstalk—particularly with key immune populations (NK cells, T cells, tumor-associated macrophages (TAMs), MDSCs, dendritic cells (DCs))—is essential. This knowledge will establish a theoretical foundation for developing exosome-targeted immunotherapies and guide the design of precise therapeutic interventions.

### 3.5 Exosome-mediated resistance to anticancer therapies

Drug resistance development poses a major challenge in cancer therapy. Exosomes, as critical mediators of intercellular communication, play essential roles in conferring tumor drug resistance (94). Nanoparticle tracking analysis has shown that docetaxel (DTX)-resistant DU145 PCa cells release a significantly higher quantity of exosomes compared to their DTX-sensitive counterparts (95). Clinical studies evaluating the protein content of exosomes derived from PCa patients have confirmed that PCa cell-derived exosomes markedly regulate tumor cell invasiveness and chemoresistance. Cumulative evidence suggests that exosome-mediated drug resistance in tumors is governed by a variety of complex molecular mechanisms. These include: (1) the direct expulsion of chemotherapeutic agents via exosomal secretion, (2) the transfer of resistance-associated cargos from resistant to drug-sensitive tumor cells through exosomal communication, and (3) the role of exosomes as decoys in immune-based therapies (94, 96). Among these, numerous studies have primarily attributed tumor drug resistance to the second mechanism, whereby exosomal transfer of molecular contents contributes to the dissemination of resistance traits.

Multiple studies have indicated that specific exosomal microRNAs play pivotal roles in mediating chemoresistance in cancer cells. For example, Corcoran et al. identified four miRNAs—miR-598, miR-34a, miR-146a, and miR-148a associated with DTX resistance in PCa using RNA expression microarray analysis. Among them, miR-34a primarily contributes to DTX resistance by inhibiting the expression of BCL-2 (97). Similarly, Li et al. reported that exosomal miRNAs, including miR-32-5p, miR-141-3p, miR-606, miR-381, and miR-429, may induce chemoresistance in PCa by modulating the androgen receptor, PTEN, and the hub gene TCF4 (T-cell factor/lymphoid enhancer-binding factor 4) (98). One study demonstrated that exosomes secreted by primary PCa fibroblasts are enriched in miR-27a, which suppresses p53 expression in PC3 cells, thereby exacerbating chemoresistance in metastatic castration-resistant PCa (mCRPC) cells (99). Additionally, exosomal miR-432-5p derived from CAFs isolated from patient-derived PCa tissues was shown to promote DTX resistance by targeting CHAC1, reducing glutathione (GSH) consumption, and ultimately inhibiting ferroptosis in PCa cells (100). Moreover, miR-423-5p in CAF-derived exosomes confers taxane resistance in PCa by suppressing TGF-β signaling and downregulating GREM2 (101).

Similarly, other exosomal components have also been implicated in mediating drug resistance in PCa. The acquisition of DTX resistance in DTX-sensitive PCa cell lines (DU145, 22Rv1, and LNCaP) has been associated with the exosomal release of multidrug resistance protein 1/P-glycoprotein (MDR-1/P-gp) (102). In another study, silencing P-gp expression in exosomes significantly reduced DTX resistance in PC3 cells, suggesting that exosomal P-gp plays a critical role in mediating chemoresistance in PCa (103).Additionally, circSLC4A7, enriched in exosomes derived from resistant PCa cells, was found to promote DTX resistance via the miR-1205/MAPT axis (104). The exosomal circular RNA circ-XIAP (X-linked inhibitor of apoptosis) directly targets and inhibits miR-1182, thereby upregulating TPD52 expression and contributing to DTX resistance in PCa, which may represent a promising therapeutic target for overcoming chemoresistance (105). Tan et al. further demonstrated that exosomal circ-SFMBT2, which contains four malignant brain tumor domains, enhances DTX resistance in PCa by suppressing miR-136-5p and upregulating TRIB1 expression (106). Moreover, exosomes derived from LNCaP cells are enriched in Caveolin-1, which can confer DTX chemoresistance to recipient cells and increase their survival following radiotherapy (41). Bhagirath et al. revealed that resistance to enzalutamide and the induction of treatment-associated NED in PCa are also mediated via exosomes (81). Notably, inhibiting exosome secretion partially restored enzalutamide sensitivity in resistant PCa cells. Furthermore, enzalutamide-treated PCa cells release exosomes carrying the neuronal transcription factors BRN2 and BRN4, which drive oncogenic reprogramming of prostate adenocarcinoma toward a neuroendocrine phenotype (81).

LincROR is upregulated in doxorubicin-resistant PCa cells and undergoes hnRNPA1-dependent exosomal packaging. This facilitates chemoresistance transfer to recipient cells, promoting DTX resistance in PCa. Mechanistically, lincROR stabilizes MYH9 protein via direct interaction, activating β-catenin/hypoxia-inducible factor 1-alpha (HIF1α) signaling. These findings indicate that exosomal lincROR drives DTX resistance through a β-catenin/HIF1α positive feedback loop (107). Separately, Zhang et al. reported that CAF-secreted neuregulin 1 (NRG1) activates HER3 in tumor cells, enhancing androgen deprivation therapy resistance. Pharmacological inhibition of the NRG1-HER3 axis effectively suppresses hormone resistance development (108).

Collectively, these studies highlight exosomes as key mediators of PCa drug resistance, positioning exosome-targeting strategies as promising therapeutic approaches to overcome chemoresistance.

## 4 Exosomes in prostate cancer: tumor-suppressive roles

**Figure 3:**
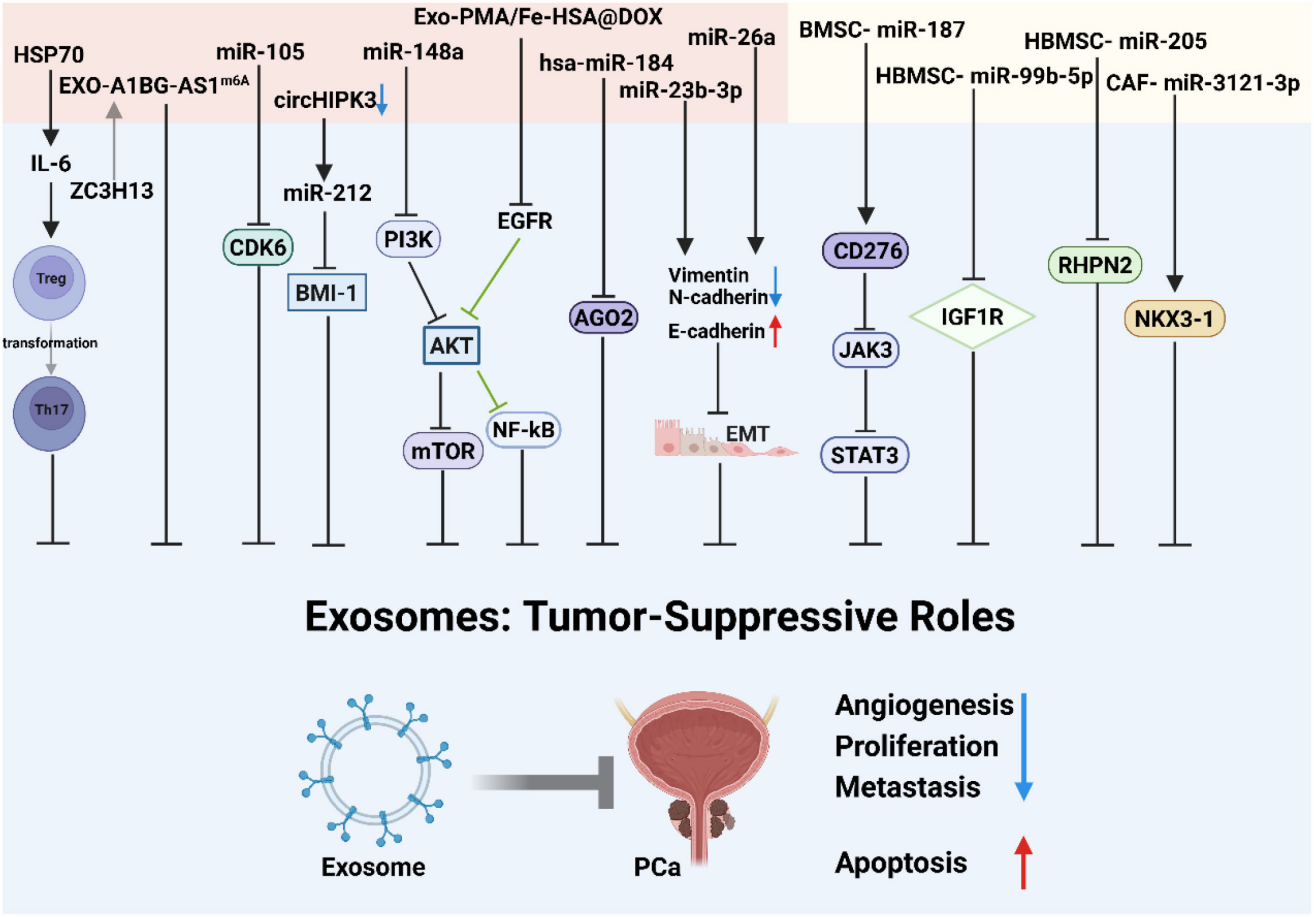
Exosomes in prostate cancer: tumor-suppressive roles. The pale red section on the left indicates exosomal components derived from prostate cancer cells, while the paleyellow section on the right represents exosomal materials from bone marrow-derived mesenchymal stem cells (BMSCs) and fibroblasts. Created with BioRender.com.

Beyond the documented detrimental roles of exosomes in PCa progression, emerging evidence supports their tumor-suppressive functions (**Figure 3**).

Exosomal hsa-miR-184 derived from the plasma of PCa patients acts as a novel negative regulator of angiogenesis by targeting Argonaute 2 (AGO2), a core component of the RNA-induced silencing complex (RISC), in human umbilical vein endothelial cells (HUVECs). Mechanistic studies demonstrate that exosome-delivered high levels of hsa-miR-184 significantly suppress AGO2 expression. This suppression consequently impairs HUVEC proliferation, migration, and *in vitro* tube formation capacity, ultimately blocking tumor-associated angiogenesis. This finding establishes exosomal hsa-miR-184 as a novel anti-angiogenic factor in PCa and highlights its translational potential as a therapeutic target for anti-angiogenic strategies (109). Exosomal miR-26a originating from the low-grade PCa cell line LNCaP significantly modulates the expression of EMT-related factors. This includes downregulating matrix metalloproteinases (MMP-2, MMP-9) and mesenchymal markers (N-cadherin, Vimentin), while concurrently upregulating the tissue inhibitor of metalloproteinase 2 (TIMP2) and the epithelial marker E-cadherin. This inhibitory effect on the EMT process ultimately results in a significant attenuation of PCa cell metastatic potential and *in vivo* tumor growth (110).Tian et al. reported that exosomes derived from PC-3 cells suppress osteoclast differentiation via downregulation of miR-148a. This suppression was manifested by reduced expression of osteoclast maturation markers integrin β3 (ITGβ3) and matrix metalloproteinase 9 (MMP-9), alongside upregulated expression of the transcription factor MAFB. This process effectively prevents PCa bone metastasis through blockade of the PI3K/AKT/mTOR signaling pathway (111). Increased circHIPK3 levels in serum exosomes of PCa patients drive oncogenesis. Functional studies show that circHIPK3 knockdown inhibits tumor growth and metastasis through miR-212/BMI-1 signaling (112). Zhou et al. identified a significant decrease in the expression of exosomal miR-23b-3p in the serum of PCa patients. Functional investigations revealed that restoring miR-23b-3p expression effectively suppressed PCa cell proliferation and invasion. Mechanistically, miR-23b-3p modulates the expression profile of EMT-related proteins by targeting specific signaling pathways. This involves upregulating the epithelial marker E-cadherin while downregulating mesenchymal markers N-cadherin and Vimentin, thereby reversing the pro-metastatic EMT phenotype (43). Research by Honeywell et al. demonstrated that exosomal miR-105 derived from prostate tumor cell lines (PC3 and DU145) potently inhibits the proliferative activity of PCa cells by specifically targeting and suppressing the expression of cyclin-dependent kinase 6 (CDK6) (113). A1BG-AS1 is a lncRNA whose stability is enhanced via ZC3H13-mediated m^6^A modification. Studies indicate that exosomal delivery and m^6^A RNA modification play crucial regulatory roles in PCa progression (53, 114–116). ZC3H13 promotes the stable expression of A1BG-AS1 by regulating its m^6^A levels. Exosomes derived from PCa cells are enriched in m^6^A-modified A1BG-AS1, which suppresses tumor progression through a ZC3H13-dependent mechanism (117). Guo et al. reported that exosomes secreted by heat-stressed tumor cells (HS-TEXs), enriched in heat shock protein 70 (HSP70), inhibit tumor growth. This occurs by triggering IL-6-mediated immunomodulation that promotes the conversion of regulatory T cells (Tregs) to T helper 17 (Th17) cells (118). Engineered nanocarriers based on cancer cell-derived exosomes have achieved tumor-specific targeted delivery. For instance, the Exo-PMA/Fe-HSA@DOX nanosystem utilizes urine exosome-mediated homologous targeting. It concurrently blocks the epidermal growth factor receptor (EGFR) and its downstream AKT/NF-κB/IκB signaling pathway, synergizing chemotherapy with photothermal therapy (chemo-PTT) to induce cancer cell apoptosis (119). Notably, a study exploring racial disparities in PCa revealed that exosome secretion was markedly increased under hypoxic conditions across multiple human PCa cell lines (including LNCaP, 22Rv1, PC-3, and PWR-1E). This adaptive response may confer a survival advantage by eliminating metabolic waste products like lactate, thereby maintaining intracellular metabolic homeostasis within tumor cells. This mechanism was particularly pronounced in African American patients, suggesting that exosome-mediated metabolic reprogramming exhibits racial specificity in PCa progression (120).

Mesenchymal stem cell (MSC)-derived exosome therapy represents a novel strategy for targeted PCa treatment. It demonstrates significant potential in modulating the tumor microenvironment and delivering functional molecules, providing a new direction for developing highly efficient and precise therapeutic regimens (121). Exosomes originating from MSCs effectively inhibit PCa progression through multi-pathway regulation. Key mechanisms include: (1) upregulating the epithelial marker E-cadherin while downregulating the EMT transcription factor Snail to reverse EMT; (2) suppressing the expression of pro-angiogenic factors VEGF-A and VEGF-C to block tumor angiogenesis; and (3) modulating the balance between pro-apoptotic genes (BAX, p53) and anti-apoptotic genes (BCL2), thereby inducing cancer cell apoptosis (122). Exosomal miR-187 delivered by bone marrow MSC-derived exosomes (BMSC-exos) directly targets the immune checkpoint molecule CD276. This interaction inhibits the activation of the JAK3-STAT3 signaling axis and its downstream transcription factor Slug, consequently blocking aggressive PCa phenotypes (123). Human bone marrow MSC (hBMSC)-derived exosomal miR-99b-5p directly targets and suppresses insulin-like growth factor 1 receptor (IGF1R) expression, thereby impeding malignant PCa progression (124). Exosomal miR-205 sourced from hBMSCs significantly delays PCa progression by targeting and inhibiting the expression of Ras homolog protein RHPN2. This mechanism suggests miR-205 holds dual value as both a potential disease biomarker and a therapeutic target (125). Exosomes secreted by human MSCs pre-labeled with superparamagnetic iron oxide nanoparticles (Venofer^®^) – a process that did not significantly alter cellular proliferation or tumor-homing capacity – are efficiently internalized by tumor cells. These labeled exosomes suppress tumor growth in a dose-dependent manner and, under exogenous alternating magnetic field-induced hyperthermia, significantly potentiate tumor cell ablation (126).

Placental stem cell-derived exosomes exhibit selective growth inhibitory effects on highly aggressive PCa cells, with their specific targeting mechanism potentially involving tumor microenvironment modulation (127). Fibroblast-derived exosomal miR-3121-3p maintains the differentiated state in androgen-sensitive PCa cells by targeting and promoting the expression of the tumor suppressor gene NKX3-1, thereby antagonizing oncogenic dedifferentiation (128).

Collectively, these studies establish exosomes as master regulators of PCa advancement. Through targeted delivery of functional non-coding RNAs (e.g., miR-26a, miR-184, circHIPK3), they orchestrate EMT, angiogenesis, immune modulation, and metabolic reprogramming. Their inhibitory actions—such as blocking CD276/STAT3 signaling, AGO2 function, and PI3K/AKT pathways—robustly suppress tumor proliferation, metastasis, and therapy resistance. These insights unveil promising avenues for developing exosome-based diagnostic biomarkers, engineered nanotherapies, and immunocellular treatments.

## 5 Exosome-based diagnostic and therapeutic strategies for prostate cancer: current advances and clinical prospects

### 5.1 Exosomes as potential diagnostic and prognostic biomarkers for prostate cancer

Previous studies have confirmed that exosomes serve as biomarkers for cancer pathogenesis due to their distinctive biological properties (129–131). In PCa diagnostics and management, exosomes demonstrate significant utility not only as potential biomarkers but also for multiple clinical contexts including disease staging, early detection, progression monitoring, prognostic evaluation, and therapeutic response tracking. Their ubiquitous presence in diverse biofluids—such as plasma, urine, and semen (**Table 1**)—establishes exosomes as a valuable reservoir for PC liquid biopsies. This section systematically reviews advances in exosome-based diagnostic and predictive indicators for PC.

**Table 1:** Exosomes as diagnostic biomarkers for PCa.

Exosome type	Contents	Source	Expression change	Application	Ref.
miRNA	miR-21, miR-451, miR-636↓	Urine	Upregulated	Diagnosis of PC	(152)
	miR-30b-3p, miR-126-3p	Urine	Upregulated	Diagnosis of PC	(148)
	miR-21-5p, miR-574-3p, miR-141-5p	Urine	Upregulated	Diagnosis of PC	(154)
	miR-375, miR-574-3p	Urine	Upregulated	Diagnosis of PC	(155)
	miR-501-3p, miR-196a-5p	Urine	Downregulated	Diagnosis of PC	(156)
	miR-2909	Urine	Upregulated	Diagnosis of PC	(153)
	miR-141-5p, mIR-125a-5p	Plasma	Upregulated	Diagnosis of PC	(135)
	miR574, miR375, miR21	Serum	Upregulated	Diagnosis of PC	(161)
	miR-375, miR-141	Serum	Upregulated	Diagnosis of high-grade PC	(162)
	miR-1246	Serum	Downregulated	Diagnosis of high-grade PC	(163)
	miR423-3p	Plasma	Upregulated	Diagnosis of high-grade PC	(142)
	miR-1290, miR-375	Plasma	Upregulated	Prognosis in CRPC	(137)
	miR-142-3p, miR-142-5p, miR-223-3p	Semen	Upregulated	Diagnosis/prognosis in PC	(160)
	miR-654-3p, miR-379-5p	Serum	Upregulated	Treatment effect observations	(146)
	miR-125a-3p↓, miR -330-3p, miR-339-5p, miR-613	Serum	Upregulated	Diagnostic marker for bone metastasis	(143)
	miR-146a-5p, miR-24-3p, miR-93-5p	Serum	Upregulated	Diagnosis of PC	(138)
	miR-15a、miR-16、miR-19a-3p、miR-21	Plasma	Downregulated	Diagnosis of high-grade PC	(136)
LncRNA	lncRNA-p21	Urine	Upregulated	Diagnosis of PC	(157)
	SAP30L-AS1、SChLAP1	Plasma	Upregulated	Diagnosis of PC	(134)
	PCGEM1、PCA3	Urine	Upregulated	Diagnosis of high-grade PC	(164)
Protein	ITGA3、ITGB1	Urine	Upregulated	Diagnosis of PC	(158)
	Flotillin 2、TMEM256, Rab3B、 LAMTOR1、Park7	Urine	Upregulated	Diagnosis of PC	(165)
	EphrinA2	Serum	Upregulated	Diagnosis of PC	(141)
	Alpha-helical proteins ↓, beta-folded proteins	Serum	Upregulated	Diagnosis of PC	(166)
	Gamma-glutamyltransferase	Serum	Upregulated	Diagnosis of PC	(167)
	Claudin 3	Plasma	Upregulated	Plasma	(168)
	FABP5	Urine	Upregulated	Diagnosis of high-grade PC	(169)
	Survivin	Plasma	Upregulated	Diagnosis of early PC	(170)
	αvβ3 Integrin	Plasma	Upregulated	Monitoring PC progression	(144)
	TM256/LAMTOR1	Urine	Upregulated	Diagnosis of PC	(147)
	ADSV/TGM4	Urine	Upregulated	Staging PC	(171)
	CD63/GLPK5/SPHM/PSA/PAPP	Urine	Upregulated	Staging PC	(171)
	AMACR	Urine	Upregulated	Diagnosis of PC	(150)
	CD44v8-10	Serum	Upregulated	Drug resistance monitoring	(140)
	AR-V7	Plasma	Upregulated	Drug resistance monitoring	(139)
	PSMA, caveolin-1	Plasma	Upregulated	Diagnosis/prognosis in PC	(133)
	AKR1C3	Serum	Upregulated	prognosis in PC	(132)
Lipidome	Phosphatidylserine, lactosylceramide	Urine	Upregulated	Diagnosis of PC	(159)

A prospective cohort study (n=62) conducted by West China Hospital of Sichuan University on mCRPC revealed significantly elevated levels of exosomal AKR1C3 in patient plasma. Critically, survival analysis confirmed that exosomal AKR1C3 positivity serves as a significant predictor of reduced overall survival and progression-free survival. Consequently, plasma exosomal AKR1C3 detection holds promise as a novel prognostic biomarker for mCRPC patients (132). In parallel research, serum exosomes from PCa patients (n=39) exhibited marked overexpression of membrane proteins PSMA and caveolin-1 compared to those with benign prostatic hyperplasia (BPH) (n=33). This finding suggests that circulating exosomal PSMA and caveolin-1 quantification may enable early PCa diagnosis and prognostic assessment (133). Furthermore, a study of 34 PCa patients demonstrated substantially higher expression of lncRNAs SAP30L-AS1 and SChLAP1 in plasma exosomes versus controls. ROC analysis established that both lncRNAs individually distinguish PCa from BPH with high efficacy, while combining them with PSA further enhanced diagnostic accuracy (134). Collectively, SChLAP1 and SAP30L-AS1 represent promising diagnostic biomarkers, with their overexpression potentially correlating with tumor aggressiveness and disease progression.

In a screening cohort of 31 PCa patients, plasma exosomal RNA analysis via qRT-PCR identified miR-125a-5p and miR-141-5p as potential prognostic biomarkers (135). Concurrently, a multimodal approach combining qRT-PCR detection of downregulated serum exosomal miRNAs (miR-15a, miR-16, miR-19a-3p, miR-21) with ¹H-NMR metabolomic profiling successfully distinguished mCRPC patients from benign prostatic hyperplasia (BPH) cases (136). Further supporting prognostic utility, Huang et al. performed plasma exosomal RNA sequencing in 23 CRPC patients, validating that elevated miR-1290 and miR-375 levels significantly correlated with reduced overall survival (137). Diagnostically, Zhang’s team established a serum exosome-based 3-miRNA signature (miR-146a-5p, miR-24-3p, miR-93-5p) for PCa detection (138). Regarding therapeutic resistance, plasma exosomal AR-V7 measured by ddPCR in 36 CRPC patients predicted hormonal therapy resistance (139), while serum exosomal CD44v8–10 mRNA overexpression in 50 docetaxel-resistant CRPC cases demonstrated diagnostic value for chemoresistance (140). Notably, Li et al. revealed significantly elevated serum exosomal EphA2 receptor levels in 50 PCa patients versus BPH/controls. Exosomal EphA2 outperformed total serum EphA2 in discriminating PCa from BPH, indicating its dual role as the primary functional circulating EphA2 fraction and a dynamic biomarker for disease monitoring (141).

qRT-PCR profiling of serum exosomal miRNAs revealed miR-423-3p as the most significantly dysregulated species across multiple validation phases: an initial cohort (108 treatment-naïve PCa *vs*. 42 CRPC patients), an independent replication cohort (30 treatment-naïve *vs*. 30 CRPC), and 36 non-CRPC controls. Its expression consistently demonstrated robust correlation with CRPC status, supporting its potential as an early predictive biomarker (142). Concurrently, Lu et al. identified blood exosomal hsa-miR-125a-3p, -330-3p, -339-5p, and -613 as bone metastasis-specific signatures through integrated analysis of 10 metastatic PCa samples and the GSE26964 public dataset (143). Furthermore, exosomal αvβ3 integrin facilitates dynamic disease progression monitoring (144), while combined assessment of miR-1290, miR-375 (137), and AR-V7 (145) enables prognostic stratification in CRPC. For therapeutic response, exosomal miR-654-3p and miR-379-5p show promise in evaluating carbon ion radiotherapy (CIRT) efficacy (146).Critically, multi-analyte panels enhance diagnostic precision: the PCA3/PCGEM1 combination improves high-grade tumor detection, whereas TM256/LAMTOR1 co-assessment increases diagnostic sensitivity. Collectively, these advances establish a translational framework for blood exosomal biomarker implementation in clinical practice (147).

Beyond blood-derived exosomes, urinary and seminal exosomes demonstrate significant diagnostic and prognostic value in PCa. In post-DRE urine samples from 14 men with elevated PSA, exosomal miR-30b-3p and miR-126-3p exhibited superior sensitivity/specificity (46.4%/88.0% and 60.7%/80.0%, respectively) for PCa detection compared to serum PSA (53.5%/64.0%), highlighting their non-invasive diagnostic utility (148). Further investigations of urine-based AMACR—a tissue-overexpressed biomarker—revealed that while early AMACR protein assays showed limited specificity (100% sensitivity/58% specificity, n=26) (149), exosomal AMACR demonstrated enhanced performance (AUC = 0.832 for PCa *vs*. BPH; AUC = 0.78 for clinically significant PCa), outperforming PSA, f/t PSA, and PSAD (150). Additionally, urinary exosomal mRNA analyses confirmed significant overexpression of ERG, PSMA, and CK19 in PCa patients (151), while exosomal miR-21, miR-451, miR-636 (152), and miR-2909 (153) showed predictive value for metastasis. Moreover, additional studies have identified significant quantitative differences in urinary exosome content between PCa patients versus those with benign prostatic hyperplasia (BPH) or healthy individuals. These differential expression patterns involve multiple molecular species including miR-574-3p, miR-141-5p, miR-21-5p (154), miR-375 (155), miR-196a-5p, miR-501-3p (156), lncRNA-p21 (157), ITGA3 and ITGB1 (158), as well as lipid components such as phosphatidylserine and lactosylceramide (159). These collective findings establish the substantial diagnostic and prognostic significance of urine-derived exosomes in PCa. Collectively, these findings substantiate the substantial diagnostic and prognostic value of urine-derived exosomes in PCa management. Notably, a seminal exosome-based panel combining PSA with miR-142-3p, miR-142-5p, and miR-223-3p further improved PCa diagnostic and prognostic accuracy (160). Collectively, these findings underscore the translational promise of multi-source exosomal biomarkers in precision PCa management.

### 5.2 Exosome-based therapeutic strategies for prostate cancer: clinical translation prospects

**Figure 4:**
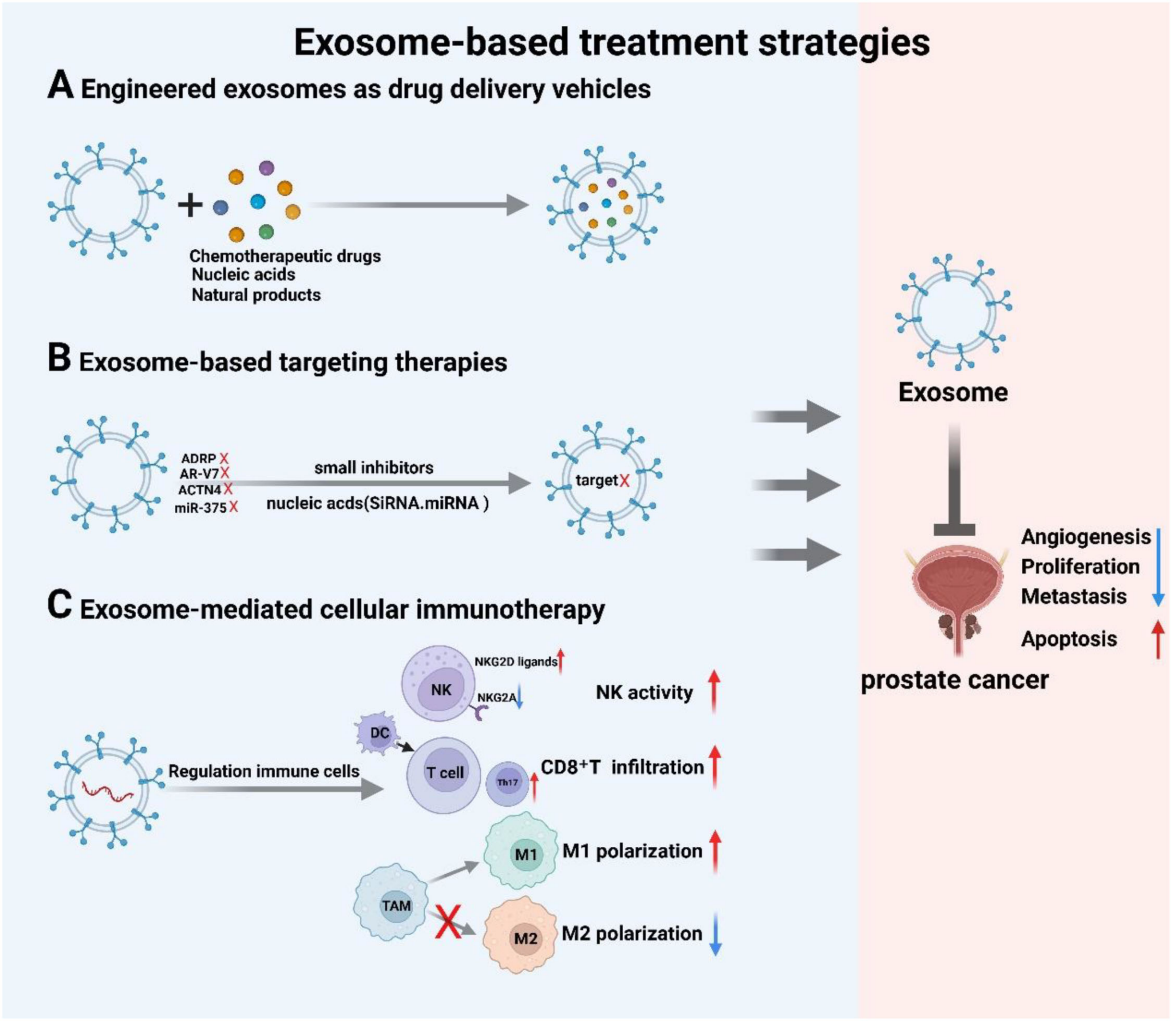
Exosome-based therapeutic strategies: **(A)** Engineered exosomes as drug delivery vehicles, **(B)** Exosome-based targeting therapies, and **(C)** Exosome-mediated cellular immunotherapy. Created with BioRender.com.

Conventional chemotherapy for cancer treatment faces significant limitations. These include suboptimal bioavailability, poor tumor targeting, induction of chemoresistance and radioresistance, potential immune-related adverse effects, and insufficient drug specificity (172). These drawbacks underscore the urgent need for novel therapeutic approaches. Exosomes present a highly promising therapeutic platform for PCa, leveraging their innate capacity to transport bioactive cargo, ease of engineering for targeted delivery, high biocompatibility, and low immunogenicity. This review summarizes three key exosome-based therapeutic strategies: (1) Engineered exosomes as drug delivery vehicles, (2) Exosome-based targeting therapies, and (3) Exosome-mediated cellular immunotherapy (**Figure 4**).

As natural nanoscale bilayer vesicles, exosomes provide an ideal platform for precision-targeted therapy by leveraging their inherent immune privilege to evade host immune clearance while utilizing their bilayer architecture for site-specific delivery of diverse therapeutic payloads—including chemotherapeutics, nucleic acids, and natural compounds (173). Their targeting efficacy is augmented through dual mechanisms: engineered surface modifications optimize ligand presentation, and intrinsic biological barrier penetration promotes lesion-specific accumulation (174–177). Biomimetic exosomal nanoplatforms effectively replicate tumor-derived exosome functions, exemplified by Vázquez-Ríos et al. drug-loaded system demonstrating high targeting precision (178). In PCa models, autologous exosomes encapsulating paclitaxel selectively enhanced cytotoxicity against LNCaP and PC-3 cells (179), while anti-PSMA peptide-functionalized exosome-mimetics showed superior targeting toward PSMA-positive lineages (LNCaP, C4-2B) versus unmodified controls *in vitro* and *in vivo* (180). Exosome-mediated delivery of tumor suppressor miR-143 significantly inhibited PC-3M-luc proliferation (181), MSC-derived exosomes transporting miR-let-7c suppressed migration and proliferation in CRPC (182), and engineered exosomes delivering SIRT6 siRNA silenced this oncogenic factor to curb metastasis (69). Immunotherapeutically, interferon-γ-anchored exosome vaccines developed by Shi et al. activated immune-mediated clearance of tumor-derived exosomes, markedly inhibiting murine tumor progression and extending survival (183). A breakthrough composite carrier—CEXO@ZIF-8/DOX, integrating zeolitic imidazolate framework-8 (ZIF-8)-encapsulated doxorubicin core with cucurbit-derived exosome-mimetic nanoparticle (CEXO) shell—achieved precise targeting, selectively inducing cell cycle arrest and apoptosis in PC-3 cells while significantly suppressing tumor growth with manageable systemic toxicity, thereby establishing an innovative exosomal delivery paradigm (184). Collectively, exosomes emerge as a transformative delivery platform for chemotherapeutics, therapeutic nucleic acids, and natural products, capitalizing on engineerable targeting, exceptional biocompatibility, and versatile cargo-loading capabilities.

Exosomal components demonstrate significant therapeutic targeting potential in PCa. Ishizuya et al. identified substantial enrichment of actin-4 (ACTN4) in serum exosomes from CRPC patients through proteomic analysis. Subsequent RNAi-mediated ACTN4 knockdown effectively suppressed DU145 cell proliferation and invasion, establishing exosomal ACTN4 as a novel therapeutic target for CRPC (185). Similarly, Gan et al. documented upregulated miR-375 expression in prostate cancer-derived exosomes, revealing its targeting as a promising strategy for CRPC with high androgen receptor expression (186). Clinical translational studies demonstrate that exosomal AR-V7 detection shows positivity in 39% of CRPC patients, with AR-V7-negative cases exhibiting significantly prolonged progression-free survival, indicating exosomal AR-V7 targeting as a viable therapeutic approach (139). Mechanistically, under androgen deprivation or IL-6 stimulation, exosomes derived from DU145 and LNCaP cells induce NED via the PPARγ/ADRP pathway. Elucidating the role of adipocyte differentiation-related protein (ADRP) in this process offers innovative therapeutic targets for advanced CRPC (80). Collectively, these findings establish exosomes and their cargos as pivotal targets for precision therapy in PCa.

Exosome-mediated cellular immunotherapy represents an innovative direction for PCa treatment. Research demonstrates that prostate cancer-derived exosomes suppress antitumor immunity through three immunomodulatory pathways: Lu et al. revealed that tumor exosomes upregulate the inhibitory receptor NKG2A on NK cells, significantly impairing NK cell activity (NKA). Conversely, radical prostatectomy increased exosomal NKG2D ligands while downregulating NKG2A, thereby restoring NKA—suggesting that targeting the exosome-NKG2A axis can reactivate NK cell cytotoxicity (187). Parallel studies by Peng et al. confirmed that inhibiting exosome biogenesis with GW4869 blocks M2-polarization of tumor-associated macrophages, reversing immunosuppressive microenvironments (188). Complementary work by Liu et al. showed that the P300/CBP inhibitor A485 suppresses exosomal PD-L1 secretion by inhibiting CD274 transcription, subsequently enhancing CD8^+^ T cell infiltration to convert immunologically cold tumors into hot tumors and potentiate immune checkpoint therapy (189). Exosomes derived from heat-stressed tumor cells are enriched with HSP70. By inducing IL-6-mediated immune reprogramming, these exosomes drive the conversion of Treg to Th17, ultimately suppressing tumor growth (118). Furthermore, recent research demonstrates that engineered dendritic cell-derived exosomes (DEX) loaded with the chemokine XCL1 (DEX~XCL1~), in combination with cisplatin, facilitate the recruitment of conventional type 1 dendritic cells (cDC1; 3.8-fold increase) and enhance the proportion of activated CD8^+^ T cells to 61.27%. This strategy effectively converts the tumor microenvironment from an immunologically barren (“immune desert”) state into a T cell-enriched status, representing a promising “cold tumor” conversion approach (190). Collectively, these findings establish a triple immunomodulatory axis targeting exosomes: NK cell activation, macrophage reprogramming, and T cell suppression reversal, providing a mechanistic foundation for developing exosome-based combination immunotherapies.

Collectively, exosomes demonstrate significant therapeutic advantages for PCa through precision targeting capabilities and inherent biocompatibility. These vesicles enhance tumor-specific accumulation of chemotherapeutics or nucleic acid therapeutics to improve efficacy while simultaneously remodeling the immunosuppressive tumor microenvironment via engineered strategies—activating NK cells, reprogramming macrophages, and reversing T cell suppression (**Figure 4**). Substantial progress in clinical translation is evidenced by registered trials validating diagnostic applications: exosomal microRNA-based prognostic assessment of tumor aggressiveness (NCT03911999), urinary exosome gene signature validation (NCT02702856), and plasma exosomal RNA diagnostic system development (NCT06604130). These studies provide critical evidence bridging basic research and clinical implementation. However, three core challenges impede clinical adoption: payload heterogeneity requiring improved cargo loading efficiency and batch consistency; safety concerns regarding immunogenicity of surface modifications and off-target effects; and paradoxical pro-metastatic risks wherein tumor-derived exosomes may accelerate progression via metastasis-promoting factor transfer. Future advancement hinges on standardized isolation protocols, intelligent engineering platforms, and rigorous biosafety assessments to establish engineered exosomes as transformative tools for precision PCa therapy.

### 5.3 Barriers to clinical translation of exosomes in prostate cancer therapy

While exosome-based therapeutic strategies hold significant promise, their translation from fundamental prostate cancer research to clinical application is impeded by fundamental challenges in standardization, manufacturing, and quality control. A primary obstacle is the absence of uniform standards for production and characterization. Current isolation techniques—such as ultracentrifugation, precipitation, and size-exclusion chromatography—exhibit considerable variability in yield and purity. This methodological diversity results in poorly defined final products and substantial batch-to-batch heterogeneity, which in turn hinders the establishment of consistent regulatory evaluation frameworks and approval pathways. Furthermore, achieving scalable production under Good Manufacturing Practice (GMP) conditions remains a critical bottleneck. Conventional laboratory-scale methods relying on culture flasks and ultracentrifugation are not only inefficient and variable but also inadequate for meeting the demands of large-scale clinical-grade manufacturing. Although scalable technologies like hollow-fiber bioreactors are under exploration, their process development, control, and cost-effectiveness require further optimization.

Implementing a rigorous quality control (QC) system is paramount to ensuring consistent safety and efficacy. A comprehensive QC strategy spanning the entire production workflow must be established, encompassing: (1) Process Controls: Standardization of critical parameters including cell source, culture conditions, and harvest timing; (2) Product Characterization: Quantitative analysis of the physical properties, biochemical markers (e.g., CD9/63/81, while avoiding contaminant markers like Calnexin), and functional attributes of the final exosome preparation; (3) Release Testing: Confirmation that sterility, endotoxin levels, particle concentration, and biological potency meet predefined standards. Only through such a systematic “quality by design” approach can therapeutic exosomes evolve from laboratory curiosities into clinically viable and reliably effective medicinal products.

Patient heterogeneity must also be factored into quality assurance. As key carriers of metabolic and physiological information, exosome composition and function are markedly influenced by donor-specific factors. In PCa, patient characteristics such as age, androgen levels, castration-resistant status, and the presence of bone metastases can profoundly alter the miRNA, protein, or lipid profiles of circulating exosomes. This variability underscores the necessity of incorporating patient stratification and personalized strategies in the future clinical development of exosome-based therapies.

Moreover, while engineering exosomes can enhance their targeting capability or drug-loading efficiency, it may also introduce novel immunogenicity risks (191). These risks primarily stem from: (1) residual parental cell proteins in allogeneic sources; (2) exogenously expressed engineered proteins or peptides; (3) introduced therapeutic nucleic acids or chemical drugs. To advance clinical translation, developing effective immune evasion strategies is crucial. Current approaches focus on conferring “stealth” properties through surface engineering. For instance, overexpression of human CD47 can bind to SIRPα on macrophages, delivering a “don’t eat me” signal that significantly reduces clearance by the mononuclear phagocyte system and prolongs circulation half-life (192). Other strategies, such as PEGylation or camouflage using natural cell membranes, can also partially shield immunogenic epitopes. Using autologous cell sources for EV production fundamentally avoids immune rejection, though it presents its own scale-up challenges. Undoubtedly, any therapeutic exosome product must undergo stringent preclinical safety evaluations before entering clinical trials. This includes systematic assessment of toxicity, tumorigenicity, and immunogenicity in relevant animal models, coupled with comprehensive characterization following International Society for Extracellular Vesicles (ISEV) guidelines (193). Adherence to the principle of “safety by design” is the cornerstone for the successful translation of engineered exosomes.

## 6 Summary and outlook

Exosomes exhibit a dual nature in PCa—serving both as pathogenic mediators and therapeutic vehicles. Naturally occurring exosomes accelerate disease progression by transferring oncogenic cargo and mediating immunosuppression, whereas engineered modifications transform them into precision-targeted delivery systems for chemotherapeutics, nucleic acid drugs, and immunomodulators. The central paradox in clinical translation stems from their biological complexity: while targeting modifications enhance drug specificity, endogenous exosomal components may propagate metastatic risks; although immunomodulatory functions activate antitumor responses, they may concurrently induce immune escape. Resolving this requires interdisciplinary innovation: employing gene editing to eliminate pro-metastatic factors while preserving targeting capacity, developing stimuli-responsive biomaterials for spatiotemporally controlled release, and integrating liquid biopsy monitoring with imaging technologies to establish dynamic surveillance networks. Ultimately, exosome-based therapeutic strategies represent a paradigm shift toward curative interventions for PCa patients.
